# Combining Remote-Sensing-Derived Data and Historical Maps for Long-Term Back-Casting of Urban Extents

**DOI:** 10.3390/rs13183672

**Published:** 2021-09-14

**Authors:** Johannes H. Uhl, Stefan Leyk, Zekun Li, Weiwei Duan, Basel Shbita, Yao-Yi Chiang, Craig A. Knoblock

**Affiliations:** 1 Earth Lab, Cooperative Institute for Research in Environmental Sciences (CIRES), University of Colorado Boulder, Boulder, CO 80309, USA; 2 Institute of Behavioral Science, University of Colorado Boulder, Boulder, CO 80309, USA; 3 Department of Geography, University of Colorado Boulder, Boulder, CO 80309, USA; 4 Spatial Sciences Institute, University of Southern California, Los Angeles, CA 90089, USA; 5 Information Sciences Institute, University of Southern California, Marina del Rey, CA 90292, USA; 6 Department of Computer Science & Engineering, University of Minnesota, Minneapolis, MN 55455, USA

**Keywords:** urbanization, long-term settlement patterns, built-up land data, global human settlement layer, historical maps, topographic map processing, data integration

## Abstract

Spatially explicit, fine-grained datasets describing historical urban extents are rarely available prior to the era of operational remote sensing. However, such data are necessary to better understand long-term urbanization and land development processes and for the assessment of coupled nature–human systems (e.g., the dynamics of the wildland–urban interface). Herein, we propose a framework that jointly uses remote-sensing-derived human settlement data (i.e., the Global Human Settlement Layer, GHSL) and scanned, georeferenced historical maps to automatically generate historical urban extents for the early 20th century. By applying unsupervised color space segmentation to the historical maps, spatially constrained to the urban extents derived from the GHSL, our approach generates historical settlement extents for seamless integration with the multitemporal GHSL. We apply our method to study areas in countries across four continents, and evaluate our approach against historical building density estimates from the Historical Settlement Data Compilation for the US (HISDAC-US), and against urban area estimates from the History Database of the Global Environment (HYDE). Our results achieve Area-under-the-Curve values > 0.9 when comparing to HISDAC-US and are largely in agreement with model-based urban areas from the HYDE database, demonstrating that the integration of remote-sensing-derived observations and historical cartographic data sources opens up new, promising avenues for assessing urbanization and long-term land cover change in countries where historical maps are available.

## 1. Introduction

By 2050, 68% of the human population is projected to live in urban areas [1]. The increasing urbanization and related processes such as rural-urban migration, socio-economic changes, and land consumption are drivers of issues such as transportation congestion and increasing pollution, posing unprecedented challenges for urban planners and policymakers. In order to make our cities more sustainable, efficient, and resilient to increasingly occurring extreme weather events, natural hazards, and climate-change-related phenomena, a thorough understanding of the long-term development trajectories of urban areas is indispensable for urban planners and policymakers. However, spatially explicit data on the size and structure of urban areas (and their changes over time) are typically derived from remote-sensing-based earth observation data, and thus rarely available prior to the 1970s. This shortcoming severely limits our knowledge of historical urban-spatial development, and forces researchers to rely on an observational window of approximately 40 years for retrospective assessments [2-4] and to establish future projections of urban land [5]. Hence, researchers studying long-term historical (urban) land development either rely on model-based approaches (e.g., [6,7]), on alternative data sources such as property data [8-11], or on the use of historical maps [12-16] that are mostly constrained to relatively small areas, such as specific cities, regions, or countries, often involving manual digitization work. This research explores the use of historical maps more specifically, as many countries have some form of historical map series, which can be used to derive knowledge related to urban areas prior to the 1970s. Integrating such spatial data with existing global remote-sensing-based settlement layers facilitates building temporally extended depictions of historical urban development for any region in the world for which such map archives have been established. This kind of integration framework is presented herein.

Recent advances in automated georeferencing [17,18], cloud-based data storage technologies, and map acquisition methods have catalyzed the availability of large historical topographic map collections to the public, often holding thousands of (georeferenced) digital raster datasets, such as in the US [19,20], the UK [21], Switzerland [22], or international collections [23-26]. Moreover, advances in the automated processing of historical maps have opened new avenues for the efficient acquisition [27] and mining of large volumes of historical maps, and for the detection, recognition, and conversion of historical map content into digital, machine-readable data formats [28-31].

Such recent efforts include the mining of (historical) map collections by their content or associated metadata [32-37], automated georeferencing [18,38-40] and alignment [41,42], text detection and recognition [43-45], and the extraction of thematic map content, often involving (deep) machine learning methods, focusing on specific geographic features such as forest [46], railroads [33,47], road network intersections [48,49] and road types [50], archeological content [51] and mining features [52], cadastral parcels boundaries [53,54], wetlands and other hydrographic features [55,56], linear features in general [57], land cover/land use [58], urban street networks and city blocks [34], building footprints [13,59,60], and historical human settlement patterns [61-63]. Other approaches use deep-learning-based computer vision for generic segmentation of historical maps [64,65], generative machine learning approaches for map style transfer [66,67], or attempt to mimic historical overhead imagery based on historical maps [68].

Many of these approaches have been tested on maps dating back to the late 1800s or even earlier but are commonly evaluated on relatively small spatial extents only. Thus, it remains unknown how such methods perform for large-scale information extraction from large volumes of historical maps, covering large spatial extents, stretching across different time periods, cartographic designs, or map scales. As a consequence, researchers have begun to develop historical map processing frameworks for large-scale data mining and extraction of heterogeneous information in a robust, feasible, and efficient manner [33].

Herein, we propose such a framework, applied to the extraction of historical urban extents. More specifically, we use urban extents from the Global Human Settlement Layer (GHSL) [69] to narrow down the regions in which urban areas in the historical maps likely occur. The GHSL data product contains the first, high-resolution settlement layer (i.e., on a 30 × 30 m grid) consistently enumerated at a global scale for the time period from 1975 to 2014 [70]. The framework presented herein combines historical maps with remote-sensing-derived settlement layers in order to extend the GHSL retrospectively (i.e., to time periods before 1975) and is, to our knowledge, the first study that analytically combines signals obtained from historical topographic maps with remote-sensing-derived data for urban change analysis. Herein, we aim to answer the following research questions:

1. Is the integrated use of signals from historical maps and from remote sensing data beneficial for the field of urban analysis?
2. What are the challenges and the requirements for an analytical framework in order to be used for the joint analysis of historical maps and remote sensing data?
3. How do the extracted urban areas from historical maps agree with other spatial-historical datasets?

Our method makes use of a back-casting strategy. While the term back-casting often refers to a specific planning strategy working backwards from a desired future state [71], the term back-casting (or hind-casting) is also used for models that generally aim to recreate past conditions [72]. The back-casting strategy employed herein spatially constrains extraction results from historical maps (from the early 1900s) to be within built-up areas from the GHSL in 1975 (see Section 2.2.2). This strategy of constraining earlier urban extents to be within the more recent and presumably more reliable area depictions [73] is commonly used in multi-temporal urban monitoring [70,74-77], as opposed to temporally independent strategies, where data for each point in time is analyzed independently, allowing for the detection of bi-directional changes (“post-classification comparison”) [78]. Thus, back-casting approaches only evaluate urban growth, not shrinkage, and may yield biased results in areas affected by urban shrinkage. However, urban shrinkage occurs relatively rarely, and typically relates to population decline and housing vacancies [79], rather than manifesting in land conversion from urban to non-urban land. Hence, we expect this bias to be marginal for the presented work. We applied our method to historical maps from six cities in four continents dated between 1890 and 1960, using historical built-up property data from the Historical Settlement Data Compilation for the US (HISDAC-US) [8,80] as well as urban area estimates from the History Database of the Global Environment (HYDE) [6] to evaluate and cross-compare our results.

## 2. Materials and Methods

### 2.1. Data and Study Areas

#### 2.1.1. Global Human Settlement Layer

From the GHSL data suite, we used the GHS-BUILT Landsat version 2018 (GHS_BUILT_LDSMT_GLOBE_R2018A), which is derived from multispectral earth observation data from the Landsat sensors, mapping built-up areas in a global grid of 30 × 30 m, referenced in a spherical Mercator projection (EPSG:3857). The GHS-BUILT data product (henceforth referred to as “GHSL”) is available for four epochs (i.e., for approximately 1975, 1990, 2000, and 2014) [69,70].

#### 2.1.2. Historical Maps

Herein, we combine the remote-sensing-derived built-up areas from the GHSL (1975–2014) with urban extents extracted from a range of historical maps dated to the period between 1890 and 1960. These maps are assumed to be the earliest available detailed topographic maps created by systematic land surveying and topographic map production, and thus will allow us to maximize the observation period for each study area. As the GHSL is a globally available product, we chose six study areas in four different countries where digital historical maps were available.

**Figure 1.**
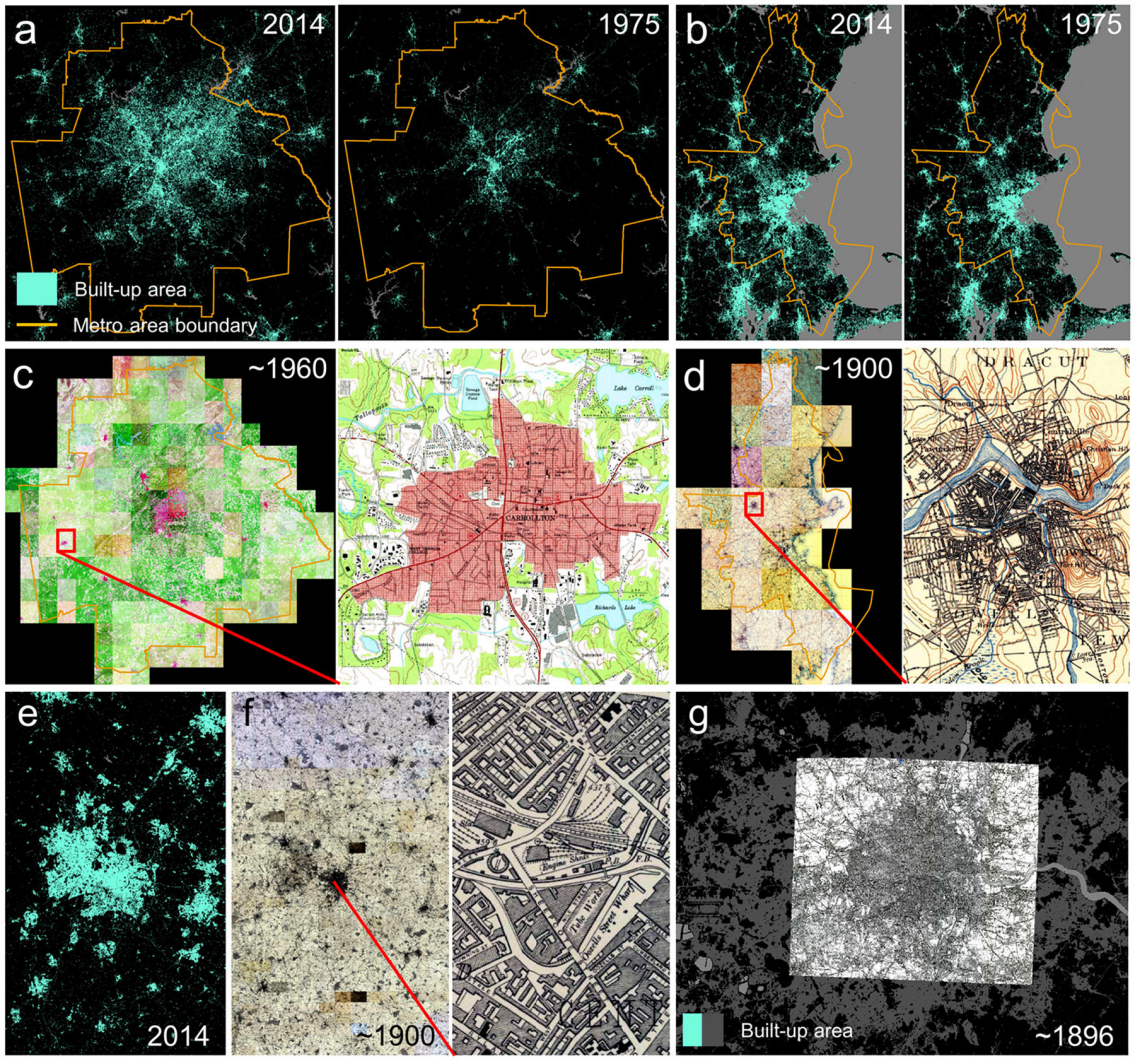
Input data for the study areas in the US and in the UK: (**a**) GHSL-based built-up area in 2014 and 1975 in Atlanta metro area (US), and (**b**) in the Boston metro area (US); (**c**) 1:24,000 historical map composite for the Atlanta metro area from approximately 1960, and (**d**) historical map composite for the Boston metro area at scale 1:62,500 from approximately 1900; (**e**) GHSL-based built-up area in 2014 in the greater Birmingham area (UK), (**f**) historical Ordnance Survey topographic map composite from approximately 1900 (approximate scale: 1:10,000), with an enlargement of a part of the Birmingham downtown area, and (**g**) historical Ordnance Survey topographic map composite from 1896 for the London area (UK; approximate scale: 1:63,000), overlaid on the GHSL 2014 built-up areas (grey).

Two study areas are located in the United States (i.e., Boston and Atlanta metropolitan areas) and cover different map scales, time periods, and map designs (i.e., 3-color print in Boston (approx. 1900, scale 1:62,500) and 5-color print in Atlanta (approx. 1960, scale 1:24,000)). These historical maps were acquired from the United States Geological Survey (USGS) historical topographic map collection (HTMC), which is a digital archive of >190,000 scanned and georeferenced topographic maps created between 1884 and 2006 [19]. The HTMC is available via the Amazon Web Services (AWS) S3 cloud data storage infrastructure [81]. The USGS-HTMC maps used herein consist of a composite of individual map sheets (see Section 2.2.1) (Figure 1a-d).

**Figure 2.**
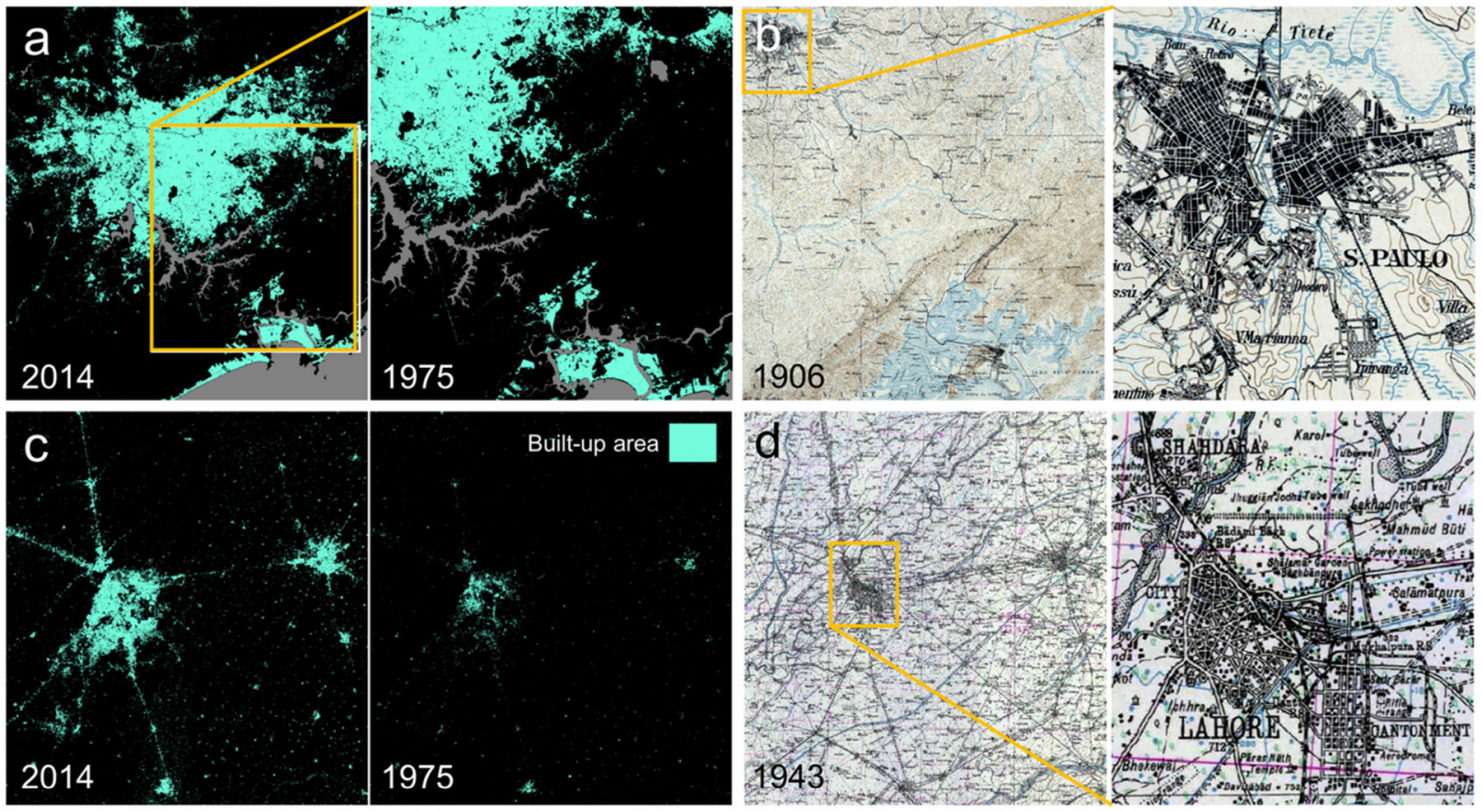
Input data for the study areas in South America and Asia: (**a**) GHSL built-up areas in 2014 and 1975 for the greater Sao Paulo area (Brazil), including the coastal city of Santos, (**b**) historical topographic map from 1906 (scale: 1:100,000) covering the same area, (**c**) GHSL built-up areas in 2014 and 1975 for the Lahore (Pakistan) and Amritsar (India) region, and (**d**) historical topographic map from 1943 (approximate scale: 1:250,000).

We also chose two study areas in the United Kingdom (i.e., Greater Birmingham and London, see Figure 2e-g), for which the National Library of Scotland provides georeferenced, seamless composites of historical Ordnance Survey topographic maps [82,83]. These maps are typically 3-color maps and exhibit different map designs than the USGS HTMC maps. For example, they depict urban settlements as blocks (Figure 1f), whereas USGS-HTMC maps use individual building outlines (Figure 1d), or red-colored urban areas in maps created after 1950 (Figure 1c). Like in the US, the Ordnance Survey maps were produced at different scales: the Birmingham maps (approx. 1900) are of scale 1:10,560 (“six-inch to the mile”), whereas the London map (1896) is at a scale of 1:63,360 (“one-inch to the mile”). We obtained the Birmingham map through an automated download and mosaicking procedure (see Section “Data Availability Statement”), and the London map through manual download, mosaicking, and georeferencing.

Finally, we use a historical topographic map covering the region southeast of the city of Sao Paulo (Brazil), at scale 1:100,000 from 1906 (Figure 2a,b), and a map covering the Lahore-Amritsar region (Pakistan/India) at scale 1:254,440 from 1943 (Figure 2c,d). Both the Sao Paulo and Lahore-Amritsar maps are individual map sheets rather than composites, and needed to be georeferenced manually, using a contemporary OpenStreetMap basemap in ESRI ArcMap 10.8.

**Figure 3.**
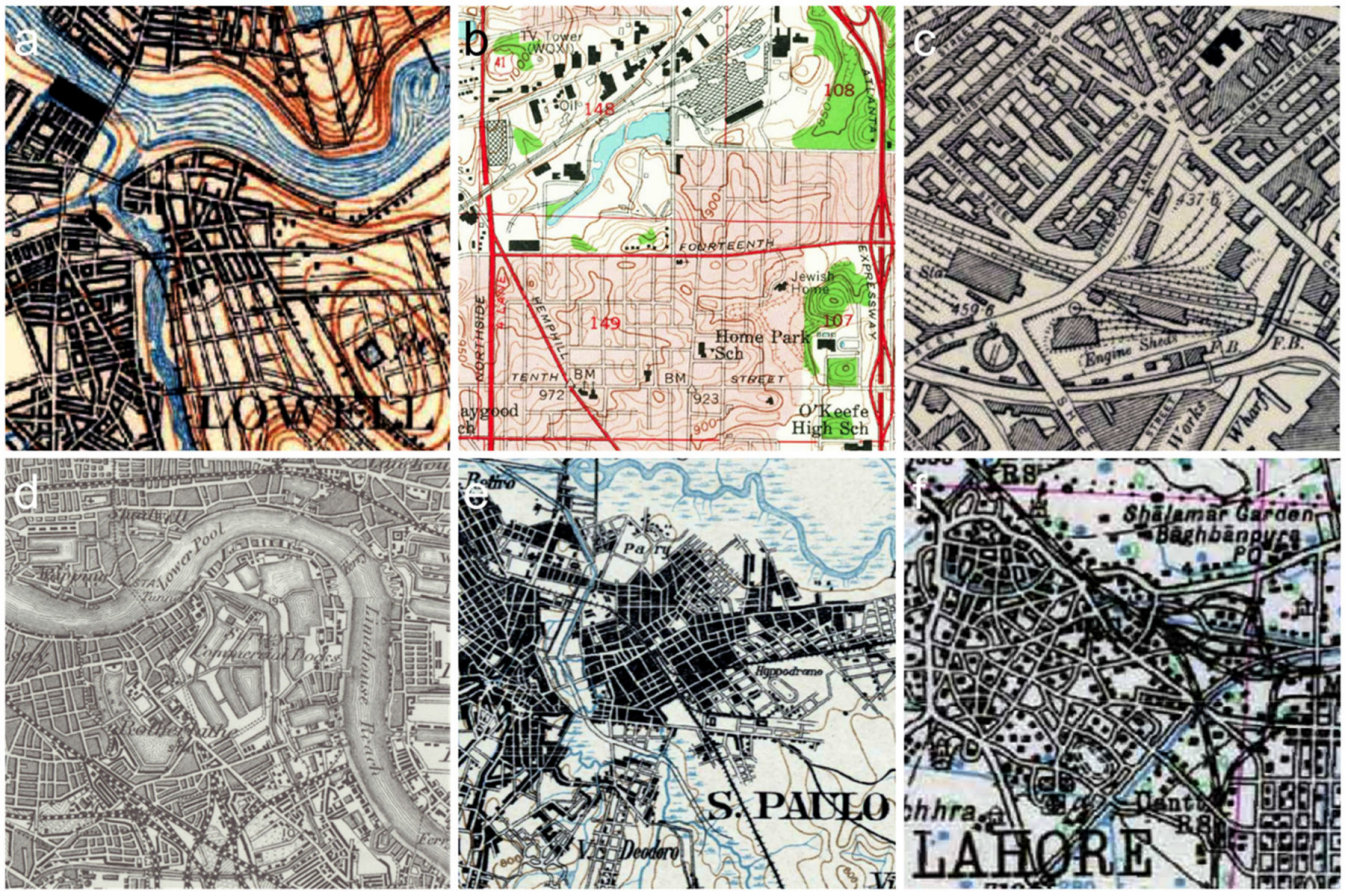
Cartographic styles used to depict urban built-up areas in the historical maps used in this study: (**a**) Boston (~1900), (**b**) Atlanta (~1960), (**c**) Birmingham (~1900), (**d**) London (1896), (**e**) Sao Paulo (1906), and (**f**) Lahore (1943).

**Figure A1.**
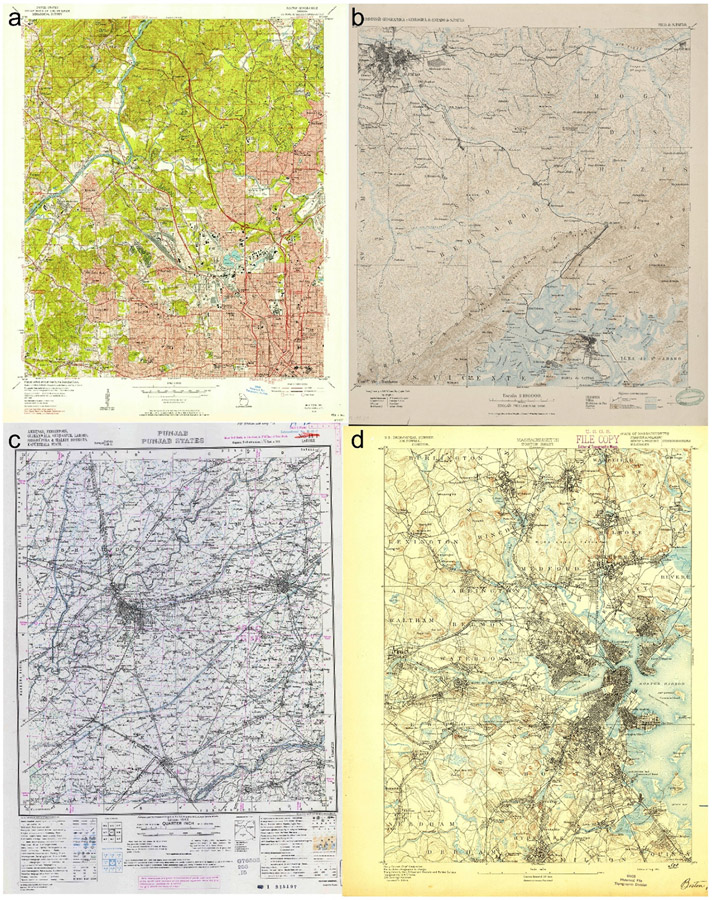
Selected scanned historical maps used in this study. (**a**) Northwest Atlanta (US) 1954, (**b**) Sao Paulo (Brazil) 1908, (**c**) Lahore/Amritsar (Pakistan/India) 1946, and (**d**) Greater Boston (US) 1893. Source (**a,d**): https://ngmdb.usgs.gov/topoview/viewer (accessed on 13 September 2021), Source (**b**): http://1886.u-bordeaux-montaigne.fr/items/show/10037 (accessed on 13 September 2021), Source (**c**): https://maps.lib.utexas.edu/maps/topo/india_253k/ (accessed on 13 September 2021).

We chose these study areas based on the availability of historical maps, and in order to cover a wide range of different map ages, map styles, map scales, and historical (colonial) settings, covering both traditionally “data-rich” (UK, US) and “data-poor” environments (Brazil, India-Pakistan), by browsing the historical map search engine available at [25]. Nevertheless, most of the maps are relatively similar with respect to their color properties (e.g., 2–3 color print), except the Atlanta maps (5-color print), which are also the most recent maps, covering the largest spatial extent. Regarding the cartographic styles used for depicting urban, built-up areas, most of the maps use either street blocks (Sao Paulo, London) or building blocks (Boston, Birmingham), while in the Atlanta maps, urban areas are depicted in a red color tone, comprising both roads and buildings, and the Lahore map uses individual (symbolic) building outlines. Figure 3 highlights these different visualization styles in detail, and Table 1 summarizes the properties of the historical maps used in this study. Moreover, Figure A1 shows selected original map sheets for four out of the six study areas.

#### 2.1.3. HISDAC-US

**Figure 4.**
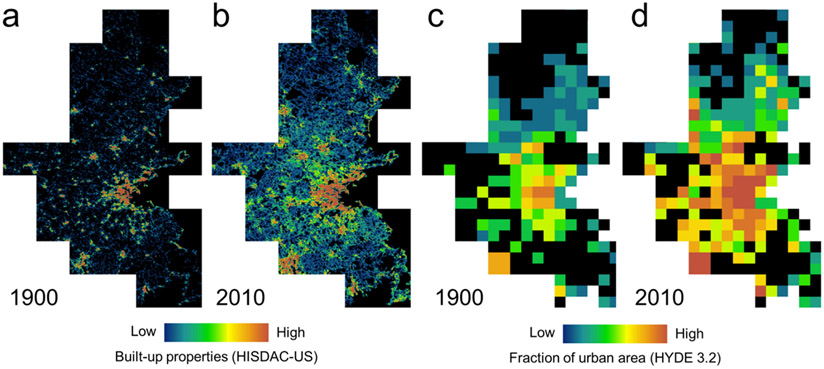
Evaluation data. Historical settlement data compilation for the US (HISDAC-US) built-up properties (BUPR) for (**a**) 1900 and (**b**) 2010, and urban area fractions per grid cell from the history database of the global environment (HYDE 3.2) for (**c**) 1900 and (**d**) 2010, all shown for the Boston metropolitan area.

Empirical data on historical urban extents are generally sparse, as remotely sensed data are typically not available prior to the 1970s. However, novel data sources such as the industry-generated property database ZTRAX (Zillow Transaction and Assessment Dataset [86]), assembled from heterogeneous county-level assessor data, holds the year-built information for large parts of the US building stock and has recently been leveraged to generate the Historical Settlement Data Compilation for the US (HISDAC-US). HISDAC-US is a fine-grained historical settlement database for the conterminous US, composed of gridded surfaces consistently enumerated in a grid of 250 m × 250 m, measuring for example, the number of built-up properties per grid cell from 1810 to 2016, which is a proxy measure for building density [9,80] (Figure 4a,b).

#### 2.1.4. HYDE Database

While the HISDAC-US data are only available for the US, we also used gridded surfaces from the HYDE 3.2 database [6], containing a global model of the fraction of urban area per 5′ grid cell, over very long time periods from 10,000 BC to 2010 (Figure 4c,d). The land use fractions in HYDE are based on “historical population, cropland and pasture statistics, combined with satellite information and specific allocation algorithms” [6]. Despite the long temporal coverage, HYDE urban area fractions do not provide much spatial detail (Figure 4c,d), and thus are not suitable for morphological analysis of historical urban areas or similar spatially explicit analyses over long time periods. Employing historical maps for such purposes may potentially fill these gaps.

Due to the model-based nature and the coarse spatial resolution, urban areas derived from HYDE are only of limited spatial compatibility when compared to the urban areas extracted at a resolution of 100 m × 100 m; however, they represent the only data source consistently available for all six study areas. Herein, we cross-compare urban area fractions from HYDE to the urban areas extracted from historical maps in all six study areas (see Section 3.4). By employing both HISDAC-US and HYDE data for cross-comparison, we expect to gain insight into the relationships of our extracted historical urban areas to historical building densities (HISDAC-US), as well as to urban land in general (HYDE).

### 2.2. Methods

**Figure 5.**
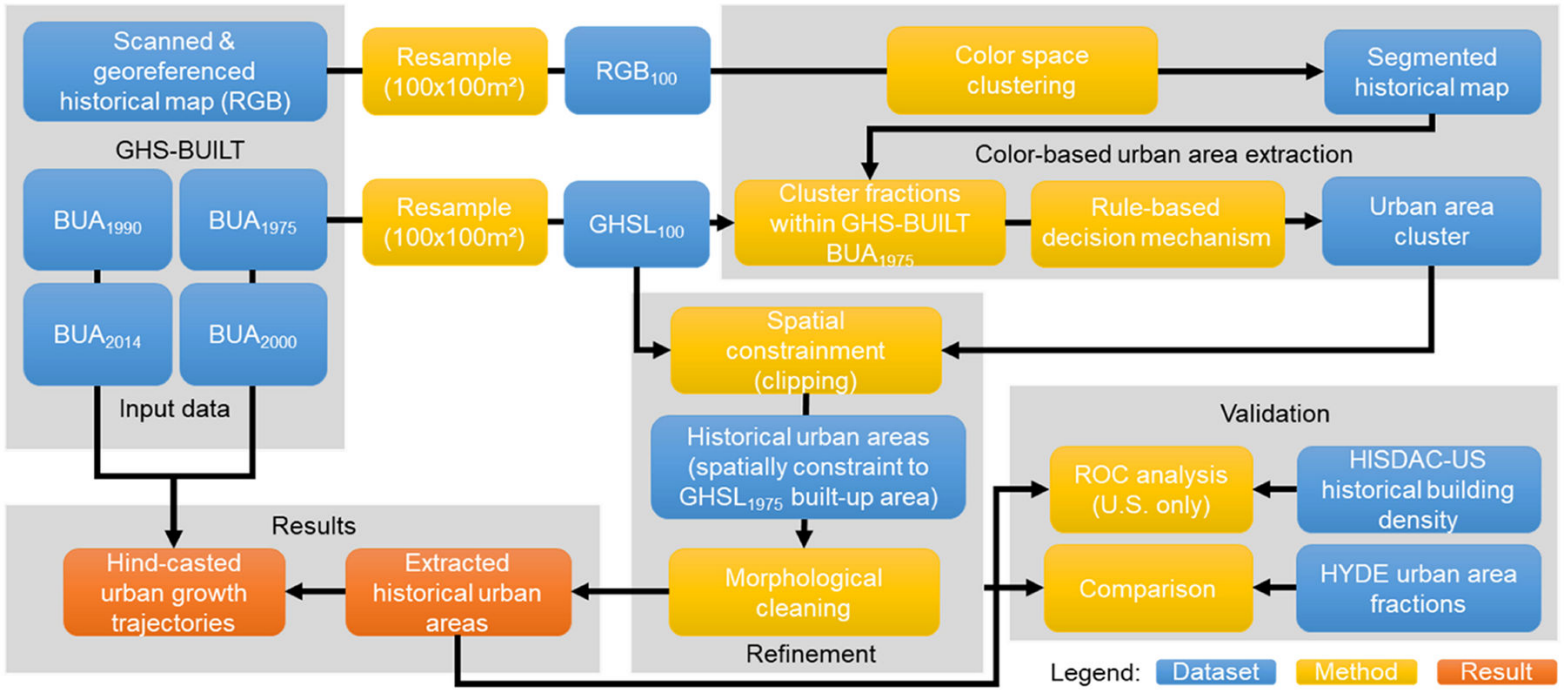
Flow diagram illustrating the historical urban area extraction and validation steps.

The methods used herein consist of the following steps: (a) preprocessing, (b) urban area extraction, (c) spatial evaluation, and (d) temporal plausibility analysis. The preprocessing includes the (automated) acquisition of historical maps, manual georeferencing for some of the study areas, and mosaicking, as well as calibration of the map data and other gridded datasets used in this study (i.e., the transformation of the datasets into common grids) (Section 2.2.1). Then, we extract urban areas from the historical maps using the GHSL as ancillary data (Section 2.2.2). This extraction process includes an unsupervised, color-based map segmentation step, the integration of map signals with built-up areas from the GHSL, a rule-based decision mechanism to identify historical urban areas, and a spatial refinement step including spatial constraining and morphological cleaning (Figure 5). We evaluate the resulting historical urban areas *across space*, by quantifying the spatial agreement with built-up density distributions from the HISDAC-US (Section 2.2.3), and evaluate our results *across time*, by assessing the agreement to the HYDE data, and by testing the plausibility of our results with respect to the GHSL-based development trajectories in each of the study areas (Section 2.2.4).

#### 2.2.1. Preprocessing

**Figure A2.**
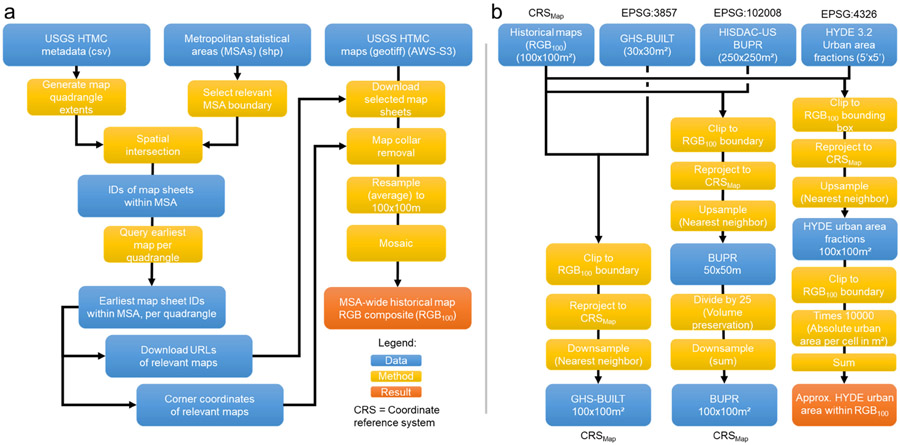
Preprocessing workflows. (**a**) The steps performed to generate spatially aggregated composites of RGB signals from historical maps for the Atlanta and Boston metropolitan areas. Panel (**b**) illustrates the reprojection and resampling steps to transform the historical map data, HISDAC-US, HYDE, and the GHSL into common grids. More specifically, the HISDAC-US based building densities are resampled from a 250 × 250 m^2^ grid to the target resolution of 100 × 100 m^2^, using an intermediate resolution of 50 × 50 m^2^, to keep additional uncertainty introduced during resampling to a minimum. Similarly, the HYDE urban area fractions, available in a 5′ x 5′ grid, are resampled to 100 × 100 m^2^, and urban area fractions are proportionally re-allocated in the target grid to keep the effects of grid cells overlapping the study area boundaries to a minimum.

Based on metadata for the USGS-HTMC (available from https://thor-f5.er.usgs.gov/ngtoc/metadata/misc/, accessed on 10 September 2021), the geographic footprints of each map sheet contained in the archive can be obtained, allowing for reconstruction of the grid (the so-called graticule) in which the USGS-HTMC map sheets are organized. For each quadrangle (i.e., grid cell of the graticule), we identified the earliest available map sheet and its scale within the boundaries of the Boston and Atlanta metropolitan statistical areas in 2010 [87] and automatically downloaded these maps from the AWS S3 archive [81]. By doing so, we obtained 33 maps for the Boston metro area, and 180 maps for the Atlanta metro area. Based on the corner coordinates available for each map sheet, we removed the map collars and generated a seamless mosaic of the maps per study area (see Figure 1c,d). The preprocessing steps for the US study areas are shown in Figure A2a.

For the study areas in the UK, we obtained the historical maps from [82,83] and mosaicked them and, in case of the London study area, georeferenced them. Individual map sheets for the Sao Paulo and Lahore study areas were manually georeferenced. All maps and map composites were then spatially aggregated by computing the RGB averages, separately per channel, within blocks of 100 m × 100 m (RGB_100_). This 100 m × 100 m grid represents the analytical unit for the subsequent analyses. Such a spatial aggregation allows for the fast processing of large amounts of maps and facilitates the integration with other gridded data.

**Figure 6.**
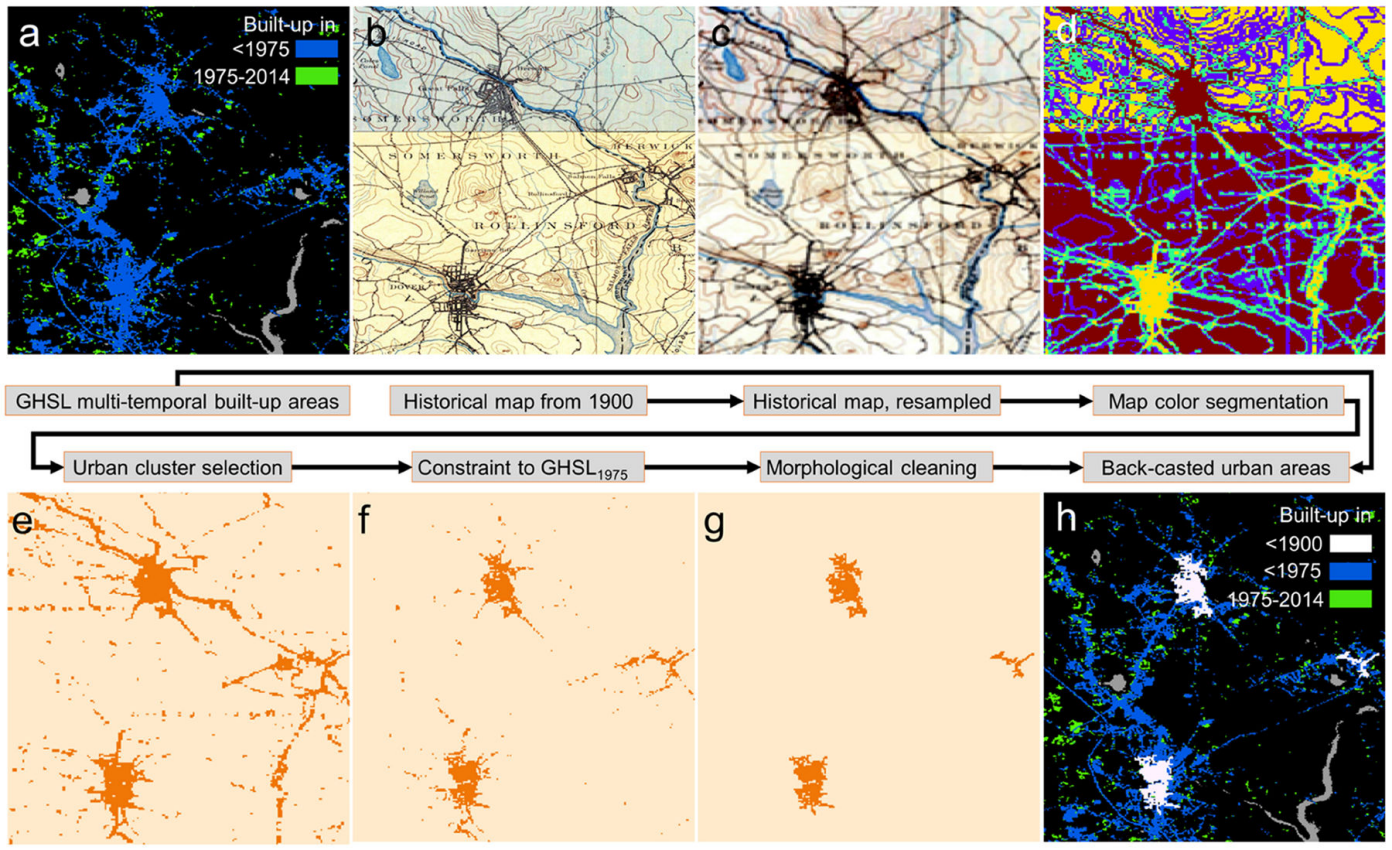
Illustrating the historical urban area extraction method using historical maps and the GHSL. (**a**) GHSL multitemporal built-up areas (resampled to 100 m × 100 m), (**b**) original historical map sheets from approximately 1900, (**c**) generated 100 m × 100 m RGB aggregates (averages per channel), (**d**) color clustering results for k = 4, (**e**) target clusters likely representing urban areas, identified by a rule-based decision mechanism taking into account the GHSL areal proportions per cluster and the cluster brightness, (**f**) target clusters within GHSL 1975 built-up areas only, (**g**) post-processed target cluster areas, and (**h**) back-casted urban areas (i.e., extracted historical urban areas integrated with the GHSL multi-temporal built-up areas).

Despite a native spatial resolution of the GHSL of 30 m × 30 m, we use a grid of 100 m × 100 m cells as our analytical unit, since such spatially aggregated RGB signals from the historical maps will allow modelling of “urban areas” in a generalized and more robust manner, regardless of how built-up structures are depicted in historical maps (e.g., individual buildings, street blocks, etc.), as a 100 m × 100 m grid cell typically extends across multiple street blocks. The resulting urban areas are assumed to encompass built-up structures, impervious surfaces, and other land use types considered to be “urban” in a broader sense, such as smaller intra-urban green spaces or similar. The effect of spatially aggregating RGB information found in the historical maps to create the RGB_100_ layers can be seen in Figure 6b,c. Using a finer analytical unit may result in oversampled results, in particular when the scanning resolution of historical maps is low. Moreover, historical maps may suffer from positional inaccuracies introduced by a range of reasons, such as inaccurate topographic measurements due to the instruments used at the time of map creation, cartographic displacements and generalization, humidity or heat-induced (non-linear) deformations of the paper map, and inaccurate georeferencing [32]. Most of these positional uncertainties are unknown and difficult to quantify. Thus, we use an analytical unit of 100 m × 100 m to obtain historical urban extents at a fine spatial granularity (as compared to coarser spatial-historical datasets such as HYDE or HISDAC-US), accounting for and potentially mitigating positional inaccuracies in the signals from the historical maps to a certain degree.

The GHSL built-up land surface as well as the HYDE urban area raster data were both clipped to the historical map extent of each study area, and resampled to create a 100 m × 100 m grid that is consistent with the aggregated map data (Figure 6a, see Figure A2b for the underlying data processing). Given the potential positional uncertainties in the historical maps, along with the vague definition of “urban areas” in general, additional uncertainty introduced during the data processing and calibration (e.g., resampling the 30 m × 30 m GHSL to the 100 m × 100 m target grid, see Figure A2b) are considered marginal.

#### 2.2.2. Urban Area Extraction

As no training data on urban vs non-urban areas are available that could be used for a supervised extraction approach, we developed a simple unsupervised method to extract the urban areas from the spatially aggregated RGB_100_ surfaces. In topographic map processing, color space clustering based on techniques such as k-means clustering [88] is commonly used for color reduction and color segmentation of scanned maps [30]. Thus, in order to reduce the color complexity, and to group the map color in meaningful ways, we performed k-means clustering on these surfaces, for a range of k ϵ [2,10].

For map composites (mosaics of individually scanned map sheets), such as in the Boston and Atlanta study areas (Figure 1c,d), we conducted a separate clustering analysis for each map sheet (Figure 6d) in order to account for potential differences in contrast or color tone (see Figure 6b,c). Moreover, we used the Elbow method [89] to identify the optimum number of clusters per study area.

Herein, we used a rule-based decision mechanism to determine which of the obtained color clusters represents the urban areas contained in the historical maps. This mechanism works as follows. We calculated the area proportion of each detected cluster within the built-up areas reported in the GHSL in 1975, aggregated to 100 m × 100 m grid cells. Assuming that the urban areas in the historical map (dated earlier than 1975) are contained within the 1975 built-up areas from the GHSL (BUA_1975_), we identified the cluster of the highest area proportion within the BUA_1975_ as the cluster likely to represent the urban areas in the historical maps. Moreover, we tested whether the average R, G, and B values of the RGB_100_ cells within that cluster were less than a given threshold value (e.g., <200). Since urban areas in historical maps are typically depicted in dark or saturated colors (black, grey, red), the use of such a simple brightness-based criterion helps to robustly identify the correct target cluster (i.e., the cluster identified as urban area).

The target cluster may still contain a considerable number of false positives (e.g., dark text elements or major roads) (Figure 6e). To reduce these artefacts, we implemented a two-step spatial refinement procedure: First, we exclude grid cells of the target cluster located outside of the BUA_1975_ extents (Figure 6f), given that this approach only detects urban growth, not urban shrinkage, which is consistent with the implemented GHSL modeling strategy and built-up concept [69]. By using the BUA_1975_ as spatial constraint, as opposed to the later epochs from the GHSL, we reduce the temporal gap between the historical map date and the constraining areas, ensuring that a maximum of false positives is eliminated. Second, we implemented a morphological post-processing strategy, removing further segments of the target cluster that are below a specific area threshold t ϵ (10, 50, 100 pixels). This is based on the assumption that settlements require a minimum size to be mapped at all. Moreover, it is unlikely that the signals of small settlements depicted in the original historical map are still detected correctly after applying the spatial aggregation to RGB_100_. Thus, small segments of the target cluster are likely to be false positives. As can be seen in Figure 6g, this method removes artefacts and retains the densely built-up urban cores of the settlements depicted in the historical map. Lastly, the grid cells identified as urban in the aggregated map layer are merged with the multi-temporal labels from the GHSL to create a temporally extended set of historical built-up land layers (Figure 6h).

#### 2.2.3. Spatial Evaluation

As described previously, a spatially explicit evaluation of the extracted historical urban extents is difficult due to the lack of reference data. The historical built-up property records (BUPR) surfaces from the HISDAC-US provide an estimate of the historical building density distributions across space in urban, but also in rural, areas and are available at a half-decadal temporal resolution. As our urban area extraction approach is assumed to be responsive to densely built-up urban areas only, a direct (i.e., binary) comparison of urban grid cells extracted from the historical map with any built-up reference grid cell (i.e., containing at least one structure) is not suitable, as it would underestimate the accuracy of our approach. Thus, we decided to carry out Receiver-Operator-Characteristic (ROC) analysis [90], which is a performance measurement tool for a (binary) classifier that yields a continuous output as a function of thresholds that are applied in order to predict class membership [91]. Herein, we use ROC analysis to test whether there is a building density threshold in the BUPR surfaces that successfully reproduces the urban/non-urban labels extracted from the historical maps. This threshold may possibly vary between maps and study areas.

We conducted such an analysis for the US study areas where HISDAC-US is available. To do so, we resampled the building densities from the BUPR layers to the RGB_100_ grids (see Figure A2b). As these historical map composites consist of individual maps produced in slightly different years (see Table 1), we created a BUPR composite that reflects the BUPR distribution in each map quadrangle in or close to the production year of the underlying historical map. For example, if a map sheet was created in 1898, we used the BUPR estimates in 1900 for the grid cells within the area covered by the map sheet.

#### 2.2.4. Temporal Plausibility Analysis

While the method described in Section 2.2.3 evaluates our results in the spatial domain, we also assessed how the hind-casted trajectories of urban area (i.e., the urban area reported in GHSL and the urban area extracted from the historical maps) agree with the trajectories extracted from the HYDE urban area dataset. In order to obtain the urban area reported in HYDE, within each study area (i.e., within the grid cells of the RGB_100_ surfaces), we clipped and resampled the HYDE urban area fraction grid to the RGB_100_ grids (see Figure A2b).

## 3. Results

### 3.1. ROC Analysis against Historical HISDAC-US Building Densities

**Figure 7.**
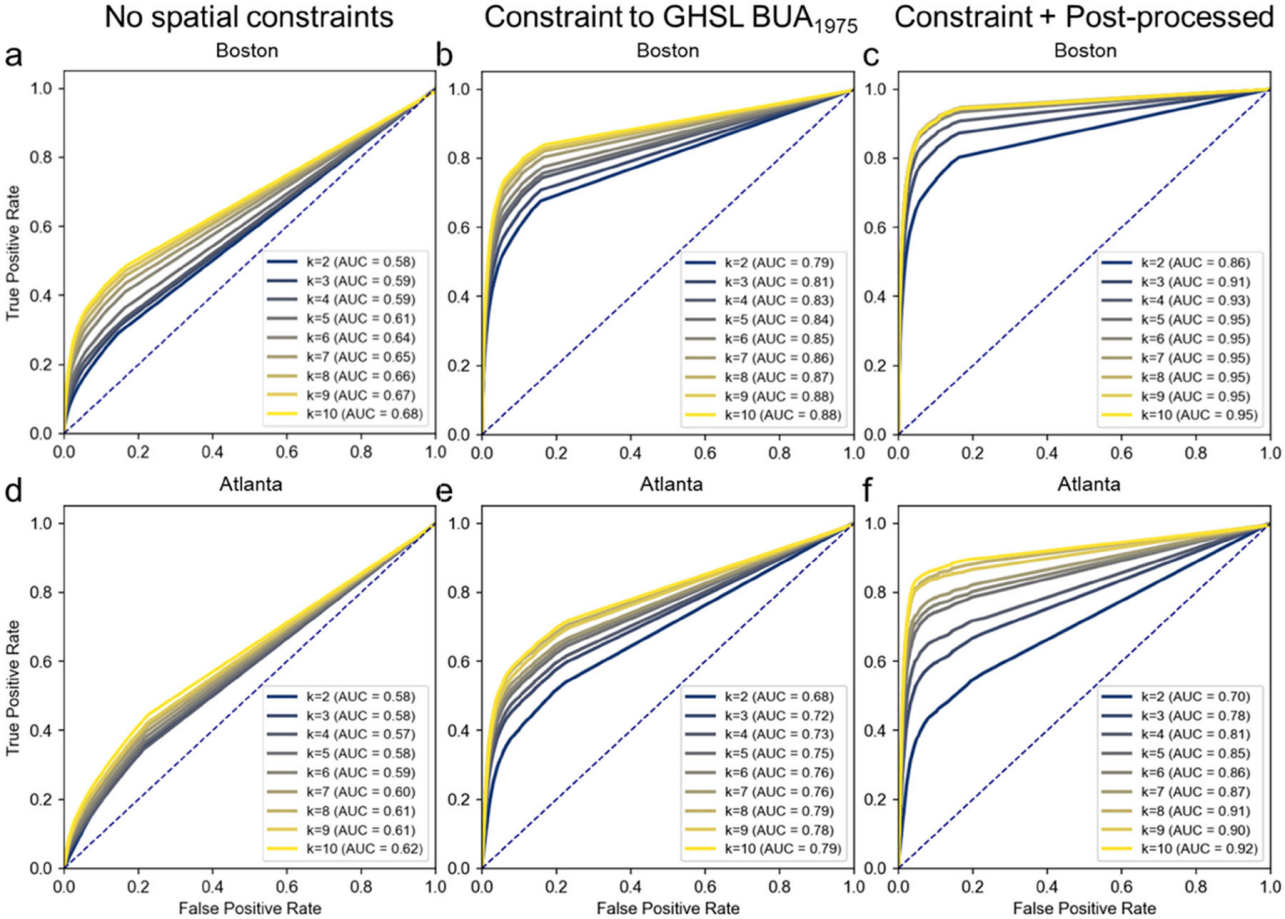
Evaluation of the map-extracted historical urban areas in the US study areas against HISDAC-US built-up property densities. (**a**) Receiver-Operator-Characteristic (ROC) plots for each clustering scenario (k from 2 to 10) without spatial constraints using GHSL 1975 built-up areas, (**b**) after applying the spatial constraints, and (**c**) after post-processing the extracted areas by removing small segments (<50 px) in Boston. Panels (**d–f**) show the ROC plots for the same scenarios in the Atlanta study area, respectively.

**Figure A3.**
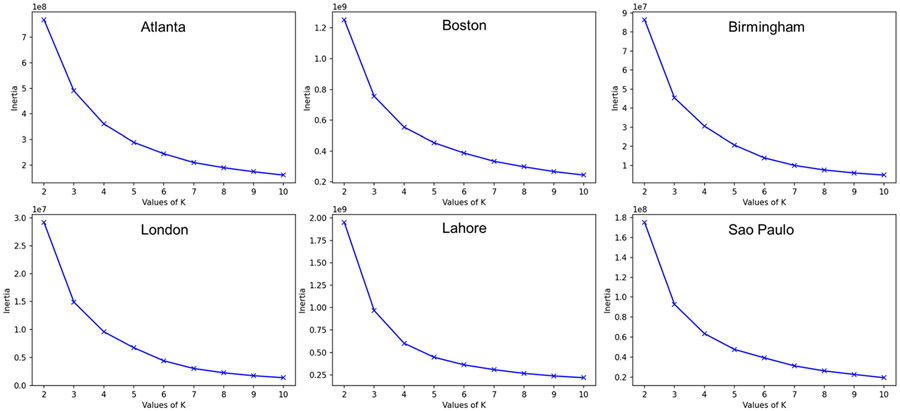
Cluster analysis results. Elbow curves based on the cluster inertia for the RGB values of the historical map raster datasets for the six study areas.

**Figure A4.**
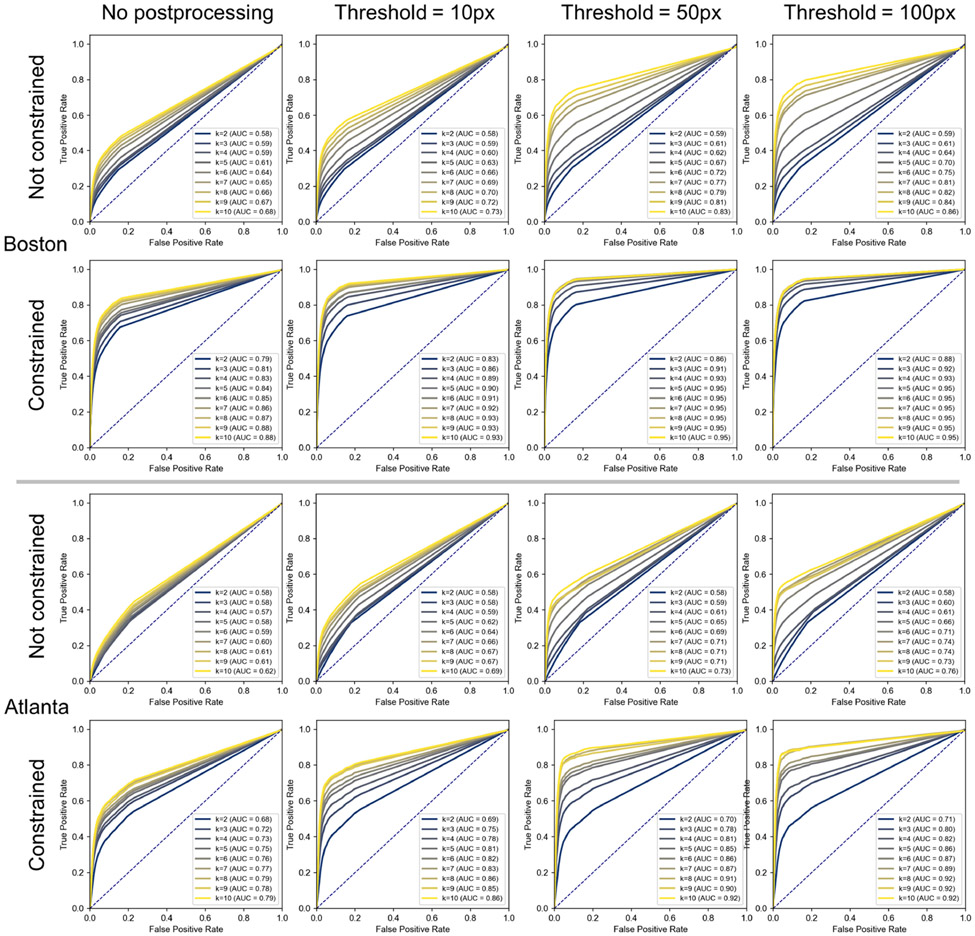
Extended ROC analysis of extracted urban/non-urban labels against the HISDAC-US BUPR estimates.

The ROC comparative analysis of extracted historical urban/non-urban labels and the historical building densities from the HISDAC-US BUPR dataset for the US study areas reveal notable effects of spatial constraining and post-processing the areas of the identified target clusters likely to represent urban areas (Figure 6). When detecting urban areas without spatially constraining them to the GHSL BUA_1975_, Area-under-the-Curve (AUC) values are low (Figure 7a,d) but increase to up to 0.88 when including the spatial constraints (Figure 7b,e). The post-processing step (i.e., removing small segments of <50 pixels) further increases the AUC to values >0.9 in both study areas. Generally, the agreement between the extracted urban areas and the BUPR estimates is higher in Boston than in Atlanta, probably due to the higher complexity of the information contained in the Atlanta maps (i.e., smaller map scale, higher number of colors and individual map sheets). The choice of the number of clusters k heavily affects the results in Atlanta, but less so in the Boston study area, where the improvement stagnates when using a k > 5 (Figure 7c). This is in line with the results of the elbow analysis, based on the inertia of the detected clusters in RGB_100_ space, suggesting that most maps (in spatially aggregated form) consist of approximately 3 to 5 main clusters (Figure A3). Herein, we use a threshold of 50 pixels for the segment removal during the post-processing step; higher thresholds do not improve the results (Figure A4).

### 3.2. Clustering Analysis

**Figure 8.**
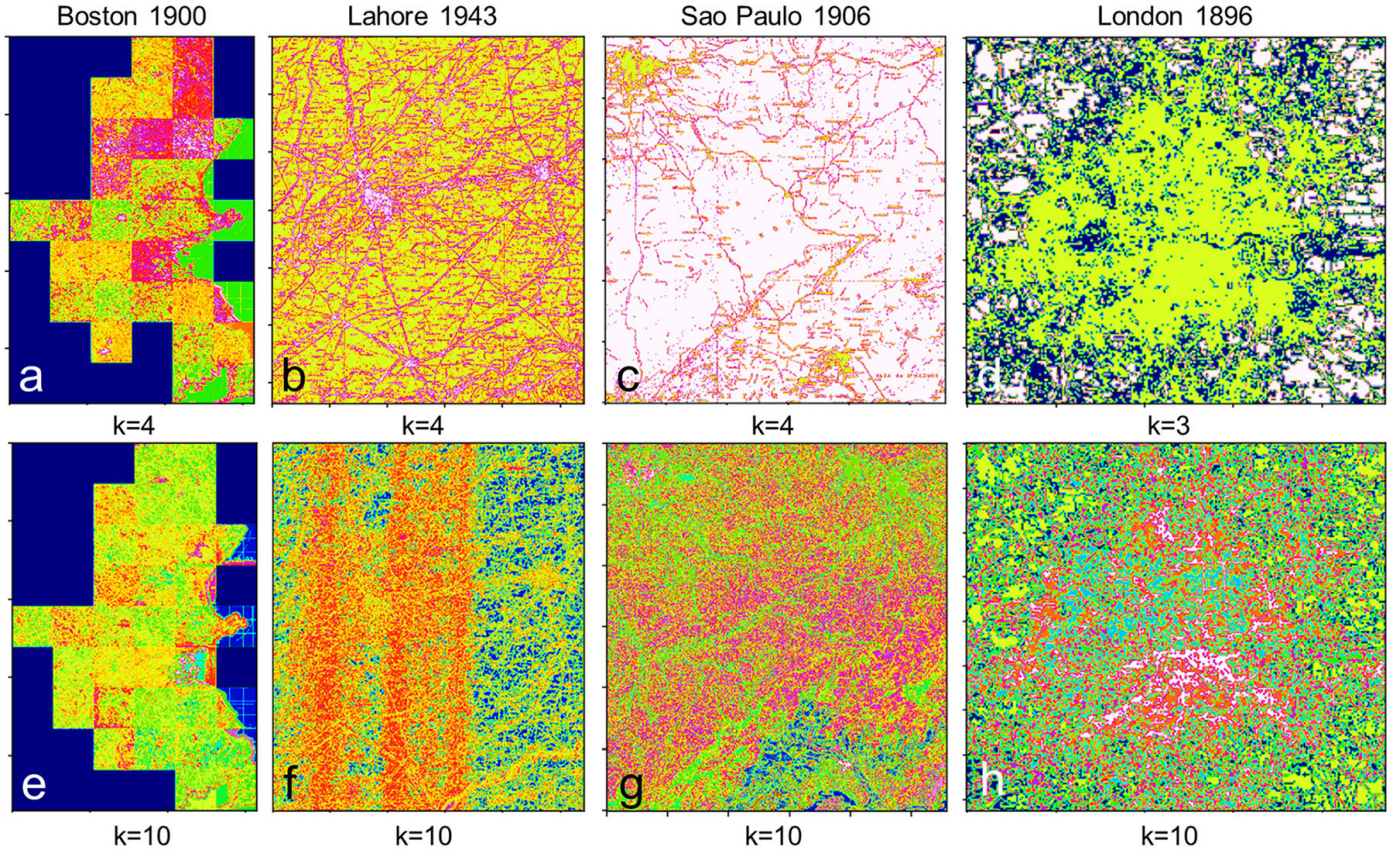
Raw color-space clustering results illustrating the effect of the number of clusters k on the spatial and semantic output granularity shown for a low k for the (**a**) Boston, (**b**) Lahore, (**c**) Sao Paulo, and (**d**) London study areas, and for a high k in (**e–h**).

**Figure A5.**
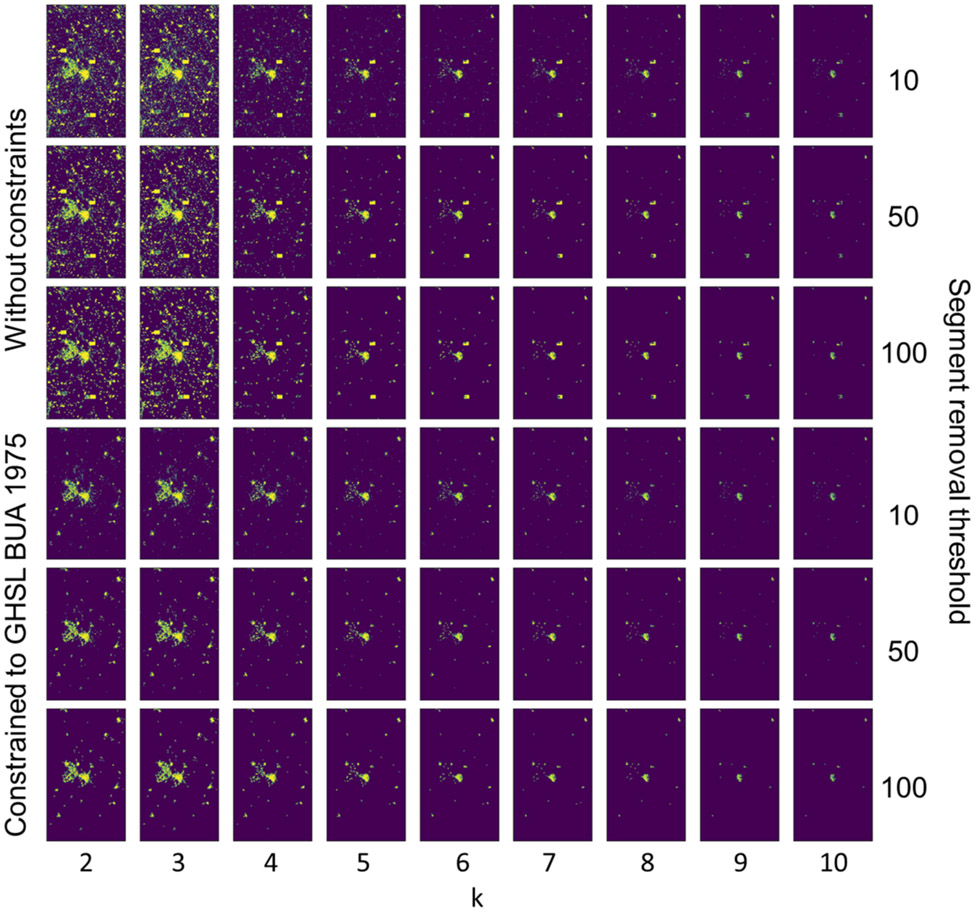
Illustrating the effects of spatially constraining the extraction results to the GHSL built-up areas in 1975, the different clustering granularity k, and the threshold used for post-processing.

While the results in the Atlanta study area seem to yield the best results for k = 10, we use k = 4 for the subsequently discussed extractions, since most maps are 3-color prints and thus are expected to perform in a similar way to the Boston study area. Thus, a granularity of k = 4 is expected to be sufficient for urban area extraction, which is shown in Figure 8a-d. However, the “mixed pixel” effects produced by the spatial aggregation of RGB information in the historical maps may cause a higher number of clusters to better characterize the density variations of specific colors (features) in the original map. For example, the clustering results using k = 10 show increasing homogeneity across individual map sheets (in the case of the Boston mosaic, Figure 8a,e), or even allow the detection of subtle scanner- or paper-induced color variations in the historical map (Figure 8f). Moreover, a higher number of clusters may even be useful to extract mountainous terrain, due to the specific RGB average values produced by densely spaced contour lines (shown in pink color in Figures 8c,f,g and A1b). An integrated illustration of the effects of spatial constraints, number of clusters, and post-processing thresholds can be seen in Figure A5.

### 3.3. Historical Settlement Extents

**Figure 9.**
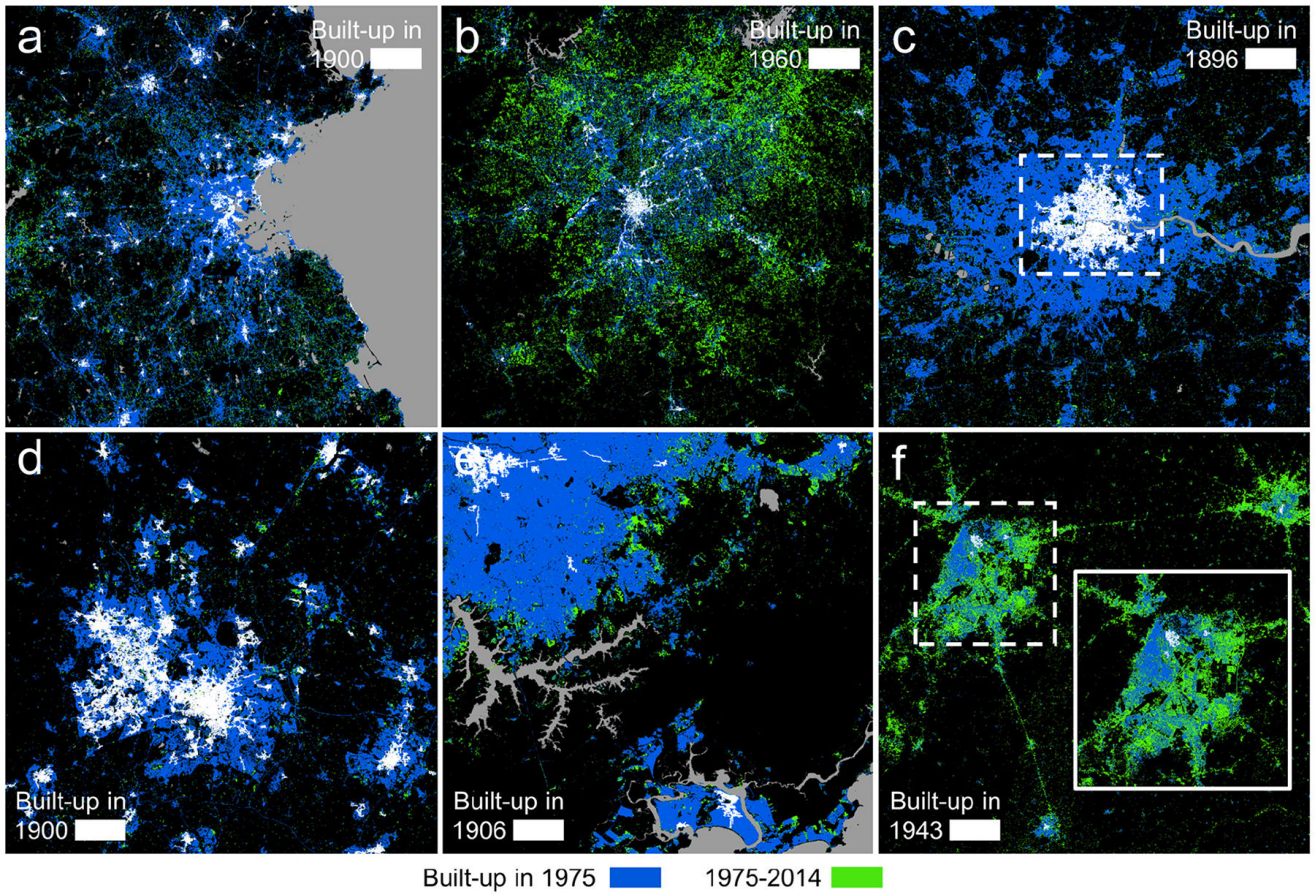
Extracted historical urban extents for all study areas. (**a**) Boston metro area, (**b**) Atlanta metro area, (**c**) London (dashed rectangle shows the study area extent), (**d**) Birmingham and surroundings, (**e**) south-east part of greater Sao Paulo area, and (**f**) Lahore-Amritsar area with an inset map of Lahore.

Finally, we show the extraction results (using k = 4 and a post-processing threshold of 50 pixels) for the six study areas in Figure 9. The extracted historical urban areas are mostly located in the center of the 1975 urban extents, which seems geographically logical, assuming concentric growth over the long term given there are no topographic constraints. These results illustrate the robustness of the decision-based identification of the urban cluster, and the effectiveness of constraining the resulting segmentation to the built-up areas from the GHSL. The visualizations in Figure 9 depict the process of urbanization that occurred prior to the remote sensing era and demonstrate the benefit of integrating remote-sensing-derived urban footprints from contemporary built-up land data and signals extracted from historical maps.

### 3.4. Cross-Comparison to HYDE and Hind-Casted GHSL Trajectories

**Figure 10.**
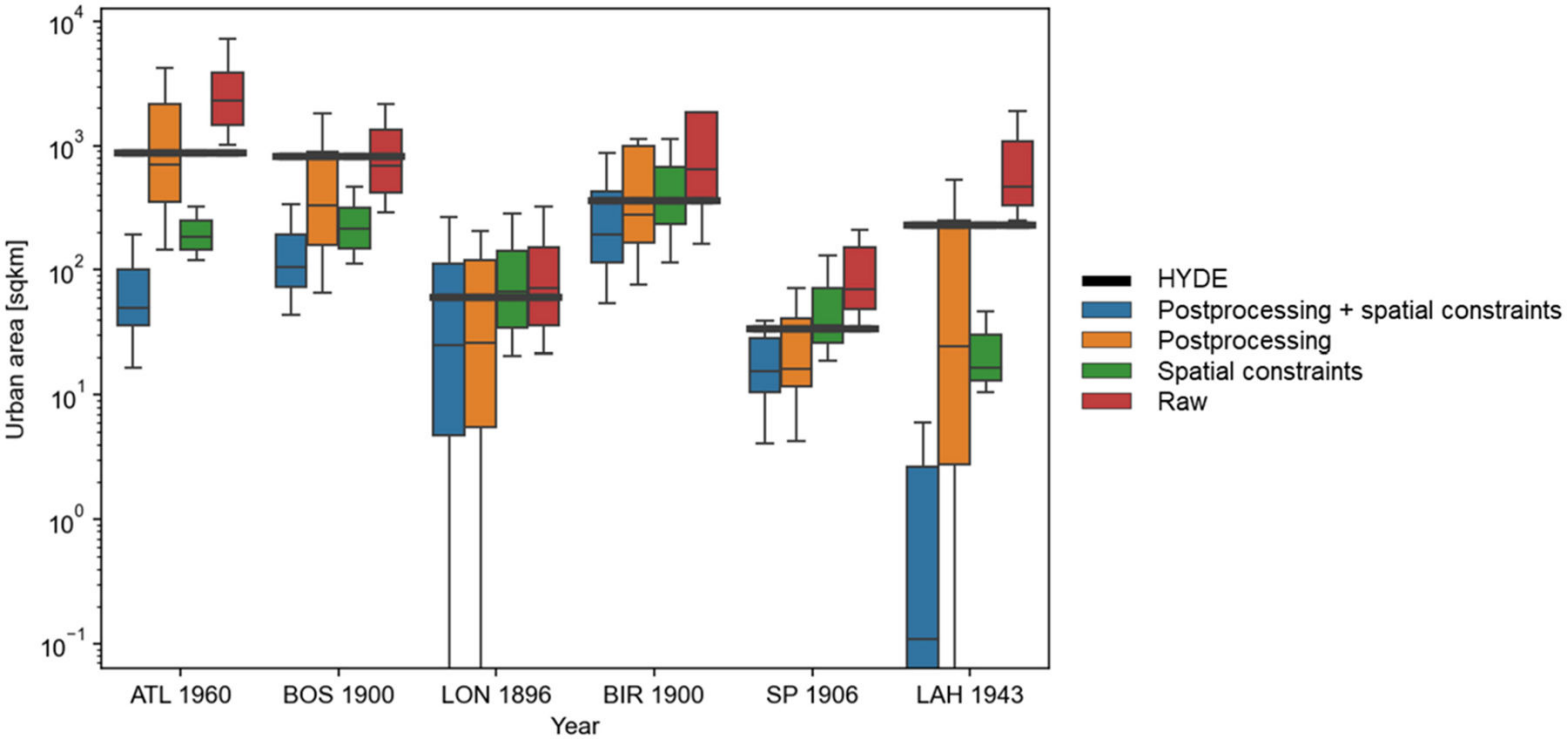
Cross-comparison to HYDE urban areas. Box-and-whisker plots illustrating the distribution of built-up areas extracted from the historical maps for all clustering scenarios, per constraint and post-processing scenario, overlaid with the urban area extracted from the HYDE 3.2 database for the respective study areas (ATL = Atlanta, BOS = Boston, LON = London, BIR = Birmingham, SP = Sao Paulo, and LAH = Lahore).

While the extraction results seem to be geographically plausible, how do they compare with the GHSL- and HYDE-based trajectories of urban areas over time? Figure 10 suggests that the extracted urban areas are largely in agreement with the trends of urban area estimated by the HYDE model, especially in the Birmingham and Sao Paulo study areas. We observe higher levels of dispersion of the extracted urban areas in London, where the extracted areas seem to be highly sensitive to the chosen post-processing parameters. Results for Lahore show higher levels of systematic deviation from the HYDE area estimate, in particular for the scenarios involving spatial constraints. This could be attributed to lower levels of data quality of the GHSL in 1975 in this area resulting in higher levels of omission of built-up areas as compared to HYDE.

**Figure 11.**
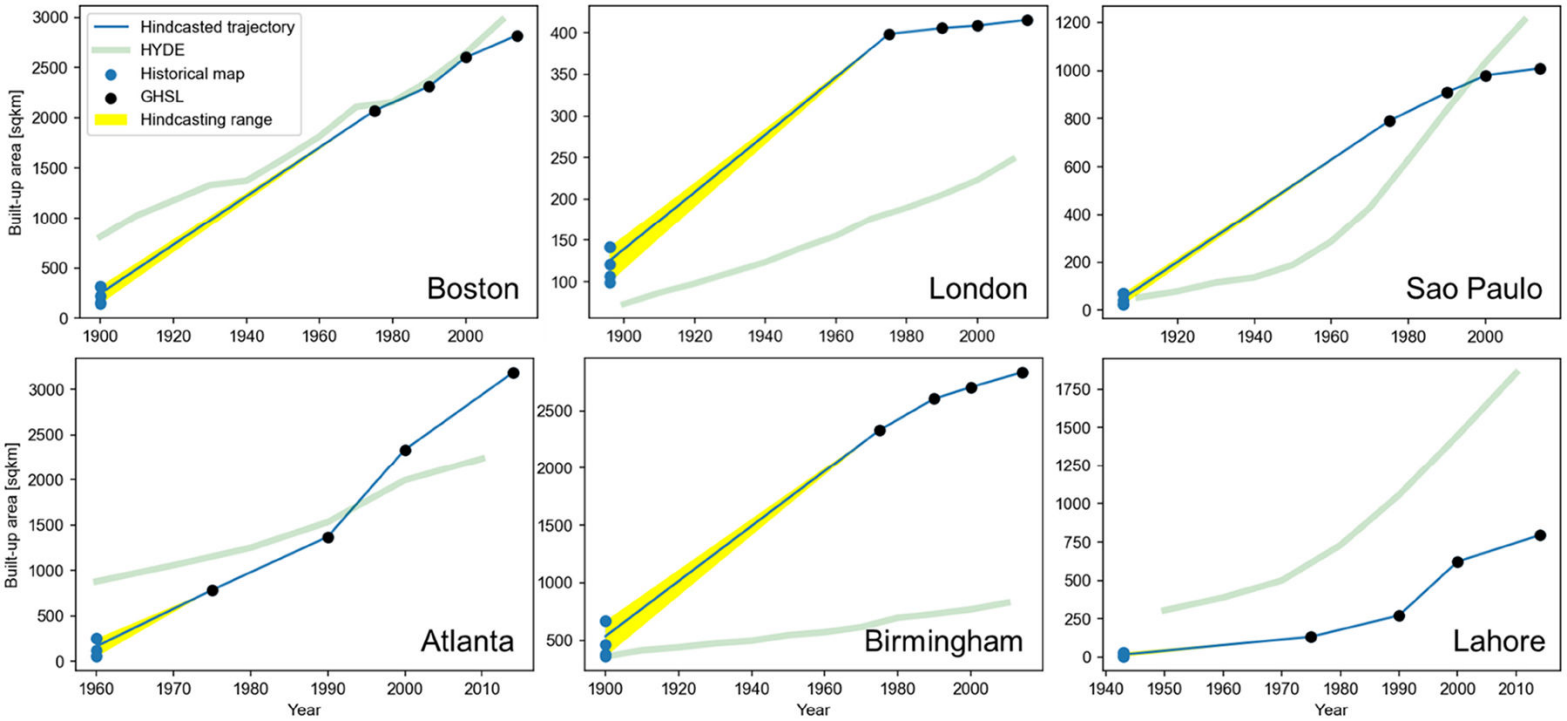
Hind-casted GHSL urban growth trajectories (extracted historical urban areas are GHSL-constraint, k = 4, averaged across any post-processing scenario) and their deviation, overlaid with HYDE 3.2 urban area trajectories. These trajectories are composed of four remote-sensing-based observations (from the GHSL) and an earlier observation obtained from the historical maps, allowing for hind-casting the GHSL-based trajectories to earlier points in time.

Lastly, we visualized the hind-casted GHSL trajectories of built-up area and overlaid them with the HYDE trajectories extracted for the same areas. Figure 11 suggests that for most cities, the hind-casted trajectory exhibits high levels of steadiness, oxcept in the London study area. A higher temporal donsity of hictorical maps would probably mitigate this effect and produce a smoother curvel. Importantly, the uncertainty of these hind-casted trajectories due to the different post-processing parameters is relatively small, and appears to be smallest in the Lahore study area (Figure 11, yellow bands). As a side note, we observe high levels of discrepancies between the GHSL built-up area and HYDE urban area estimates in some study areas, such as Birmingham. This is likely an effect of different definitions, as the GHSL includes all detected settlements (including rural settlements), whereas the urban areas in HYDE are likely to exclude those areas, but can also be attributed to the general difficulty of global models such as HYDE to estimate historical land use patterns at the regional or local level [92]. In the specific case of the London study area, this discrepancy could also be the result of edge effects due to the small study area in relation to the HYDE grid cells (i.e., 5′), which may exclude partially overlapping grid cells from the study area.

## 4. Conclusions

The work presented herein is a first attempt to create a framework that combines signals obtained from scanned, georeferenced historical maps with remote sensing data products in an integrated analytical environment. We applied this framework to the extraction and assessment of urban areas over long time periods and demonstrated how such an approach can create spatial-historical data that describe trends of long-term urban-spatial development prior to the era of remote sensing and enhance our understanding of the underlying urbanization processes.

The spatial aggregation performed on the historical maps facilitates the seamless integration with other gridded surfaces in general, and effectively reduces the spatial data volume to be processed. Thus, given the availability of georeferenced historical maps in numerous countries, this framework could be applied to systematically hind-cast the Global Human Settlement Layer, or other settlement data products, at a country scale. The presented framework represents an effective way to harvest historical maps, and thus preserve valuable knowledge that can only be found in such archival documents. Revisiting the research questions posed in Section 1, we can conclude the following:

(i) We demonstrated that signals obtained from scanned and georeferenced historical maps or whole map collections have the potential to enrich remote-sensing-based analyses (e.g., [93]) and to extend the observational window of remote-sensing-based change analysis further back in time. We applied our method to the study of long-term urban change, but similar frameworks could be employed for other applications, such as the analysis of long-term forest dynamics or other types of land cover.

(ii) Unlike remote sensing data, which is typically collected within short repeat cycles (e.g., 16 days for the Landsat 8 satellite), signals obtained from historical maps over large spatial extents may suffer from heterogeneous temporal reference. Moreover, the discussed, inherent and largely unknown positional uncertainties in signals from historical maps need to be taken into account. Thus, the choice of an appropriate analytical unit is crucial, allowing one to capture the desired spatial detail while aggregating the map signals in a way that reduces the effects of positional inaccuracies and accounts for the vague definition of the features of interest (e.g., urban areas).

(iii) Despite these challenges, the proposed analytical framework yields plausible results, that is, the extracted urban extents are largely in agreement with other spatial-historical information, and they are at a higher spatial resolution than existing spatially explicit historical data, such as HYDE or HISDAC-US.

From the ROC analysis conducted for the US study areas (Figure 6), we observe slightly higher AUC values for the Boston maps than for the Atlanta maps. This is surprising, as the Boston maps are much older (1900) than the Atlanta maps (1960). The reason is likely the number of colors used: the Boston 1900 maps are 3-color prints, whereas the Atlanta 1960 maps are 5-color prints. In addition to that, the variety of map styles in the Atlanta map composite is higher. A careful interpretation of this difference would be that the proposed method works better for map collections of homogeneous map styles with a minimum of complexity (i.e., when fewer colors are used in the historical maps), which is typically the case for older maps. This seems plausible, though it is in contrast to remotely sensed observations, where more recent data are typically more reliable than earlier measurements. However, in order to confidently formulate recommendations related to such properties, and to assess the influence of building representations in the historical maps (individual building outlines, city blocks, etc.), statistical evidence would be required by systematically analyzing a larger sample of maps from different points in time and of different map styles.

From a methodological point of view, we followed the principle of parsimony and implemented a simplistic, rule-based color clustering method to extract the features of interest, and observed satisfactory levels of performance (i.e., high levels of receptiveness with respect to historical building densities—AUC > 0.9; and consistency with model-based estimates of historical urban area) and efficiency. Future work will include the use of more advanced extraction methods, taking into account shape and textural characteristics, or more complex rule-based systems, potentially able to distinguish between high-density and low-density urban/built-up areas. The concept of contemporary spatial constraints such as delineating the results to the 1975 GHSL built-up areas, could also be incorporated into an automated training data collection procedure, which could then be used for a supervised, deep-learning approach to extract urban areas and human settlement patterns at finer spatial granularity (cf. [61]).

Concluding, we find that the presented approach appears to perform better on maps of few map colors (3-color prints) and shows lower performance on 5-color prints, even though the 3-color maps are older. When using composites of multiple individual historical maps, the method seems to work better on map composites consisting of homogeneous map styles. As our results seem plausible across all study areas, we find that the graphical quality of the underlying maps, manifested in their spatial resolution (cf. Table 1), does not affect the quality of the results drastically.

As the use of k-means color space clustering requires the manual choice of the number of clusters k, the results depend on this choice (Figure A5). Future work will include the test of alternative clustering or unsupervised classification techniques that infer the optimum number of clusters from the data [94,95], as well as the use of different color space representations [96]. Future work will also include the performance of object-based methods [57,64] as compared to the pixel-based extraction method presented herein.

Spatially explicit, historical data on built-up or urban area can enhance our understanding of the efficiency of a city over time (e.g., urban scaling analysis [97]), reduce manual labor involved in the analysis of urban-spatial trajectories [14], and allow for studying long-term urban development and land consumption processes. From a city planning perspective, however, historical information on functional properties of city elements (e.g., historical, spatially explicit urban land use, or building function) would be more beneficial than area-related information alone. While most historical topographic maps only contain limited information on land use and building functions, some map collections such as the Sanborn Fire Insurance maps [20] contain such information. However, the extraction of such information requires more complex information extraction methods, such as deep learning [98]. Future work could also focus on the extraction of functional properties from such historical maps, facilitated by contemporary remote sensing data. In a broader context, the proposed, generic tile-based framework could be expanded by using more complex feature embeddings [99] in order to estimate different kinds of retrospective land cover from historical maps.

Ultimately, such efforts create new data and insights that can inform long-term, spatially explicit land use models, such as HYDE, and can be used to improve future projections of urban land, and thus enable more informed urban planning and decision making.

## Figures and Tables

**Table 1.** Overview of the six historical maps/map composites used in this study and their properties.

City	Country	Map Type	Map Resolution [m]	Map Scale	Reference Year	Print Colors	Urban Built-Up Area Depiction	Data Source	Download & Compositing	Geo- Referencing
Atlanta	USA	Composite	2	1:24,000	1954–1969	5	Red color tone	[81]	Automated	By provider
Boston	USA	Composite	5.3	1:62,500	1885–1918	3	Building block	[81]	Automated	By provider
Birmingham	UK	Composite	2.4	1:10,560	1888–1913	2	Building block	[82]	Automated	By provider
London	UK	Composite	12	1:63,360	1896	3	Street block	[83]	Manual	Manual
Sao Paulo	Brazil	Single sheet	9.3	1:100,000	1906	2	Street block	[84]	Manual	Manual
Lahore	Pakistan	Single sheet	36.7	1:254,440	1943	2	Building outlines	[85]	Manual	Manual

## Data Availability

All data sources used herein are publicly available. The Global Human Settlement Data can be accessed at https://data.jrc.ec.europa.eu/dataset/jrc-ghsl-10007, and the HYDE dataset is available at https://dataportaal.pbl.nl/downloads/HYDE/. Individual historical maps from the USGS can be viewed and accessed at https://ngmdb.usgs.gov/topoview/viewer/, and batch downloaded from the AWS S3 repository (https://prd-tnm.s3.amazonaws.com/StagedProducts/Maps/HistoricalTopo/). Ordnance Survey maps are available as a seamless composite for viewing at https://maps.nls.uk/geo/explore/ and downloadable upon subscription from https://maps.nls.uk/projects/subscription-api/. See the reference list for the source of other historical maps used herein. The HISDAC-US data is available at https://dataverse.harvard.edu/dataverse/hisdacus. Code for USGS HTMC and Ordnance Survey historical map retrieval can be found at https://github.com/johannesuhl/histmaps and https://github.com/spatial-computing/historical_map_retrieval, respectively.
